# Mutation Scanning in a Single and a Stacked Genetically Modified (GM) Event by Real-Time PCR and High Resolution Melting (HRM) Analysis

**DOI:** 10.3390/ijms151119898

**Published:** 2014-10-31

**Authors:** Sina-Elisabeth Ben Ali, Zita Erika Madi, Rupert Hochegger, David Quist, Bernhard Prewein, Alexander G. Haslberger, Christian Brandes

**Affiliations:** 1 Austrian Agency for Health and Food Safety, Spargelfeldstrasse 191, 1220 Vienna, Austria; E-Mails: sina-elisabeth.ben-ali@ages.at (S.-E.B.A.); zmadi@gmx.net (Z.E.M.); rupert.hochegger@ages.at (R.H.); bernhard.prewein@ages.at (B.P.); 2 Department of Nutritional Sciences, University of Vienna, Althanstraße 14, 1090 Vienna, Austria; E-Mail: alexander.haslberger@univie.ac.at; 3 Centre for Biosafety–GenØk, PB 6418 Science Park, 9294 Tromsoe, Norway; E-Mail: david.quist@uit.no

**Keywords:** HRM analysis, Scorpion primer, MON810, MON88017 × MON810, GT73, genetic stability, stacked event, GMO

## Abstract

Genetic mutations must be avoided during the production and use of seeds. In the European Union (EU), Directive 2001/18/EC requires any DNA construct introduced via transformation to be stable. Establishing genetic stability is critical for the approval of genetically modified organisms (GMOs). In this study, genetic stability of two GMOs was examined using high resolution melting (HRM) analysis and real-time polymerase chain reaction (PCR) employing Scorpion primers for amplification. The genetic variability of the transgenic insert and that of the flanking regions in a single oilseed rape variety (GT73) and a stacked maize (MON88017 × MON810) was studied. The GT73 and the 5' region of MON810 showed no instabilities in the examined regions. However; two out of 100 analyzed samples carried a heterozygous point mutation in the 3' region of MON810 in the stacked variety. These results were verified by direct sequencing of the amplified PCR products as well as by sequencing of cloned PCR fragments. The occurrence of the mutation suggests that the 5' region is more suitable than the 3' region for the quantification of MON810. The identification of the single nucleotide polymorphism (SNP) in a stacked event is in contrast to the results of earlier studies of the same MON810 region in a single event where no DNA polymorphism was found.

## 1. Introduction

Since its introduction in 1996, the use of commercial genetically modified (GM) crops has drastically expanded. In the year 2013, 175.2 million hectares of GM crops were grown. Of these, less than 0.3 million hectares were cultivated in the EU (Spain, Portugal, Czech Republic, Romania, and Slovakia) to one singular maize variety (MON810) [1]. More recently, the commercialization of single events (for example MON810 maize or Roundup Ready Soy), have given way to the introduction of stacked events containing multiple transgenes [2]. Approximately 47 million hectares of stacked traits were grown in 2013, which is 3.3 million hectares more than that reported in 2012 [1]. There has been an increase in the number of approved stacked events and the number of transgenes stacked in a single plant (e.g., SmartStax™ maize, containing eight events) [2].

Authorization and compliance measures of GM food and feed require that GM events be genetically characterized and quantified. Additionally, transgene stability is important aspect to ensure food safety [3,4]. Reports on the genetic stability of genetically modified organisms (GMOs) have presented variable results. Initially, it was presumed that the production of transgenic plants involved the insertion of a single transgene and the flanking regions without further genomic disruption. However, instances of genomic alterations, including complex nucleotide rearrangements (e.g., deletions, duplications, insertions of organelle and filler DNA, and translocations) have occurred as a result of the transformation process [5,6,7,8,9,10,11,12,13]. The stability of the transgene, and gene expression as well as performance of the plant can be influenced by various factors, including host biology, genome organization, by epigenetic factors, or by nucleotide changes within the introduced DNA construct [4].

In addition to the requirement of genetic stability during GMO development, transgenic plants should demonstrate genetic stability subsequently during cultivation and propagation. Recent studies have identified the occurrence of DNA rearrangements after the transformation process and during the post-release monitoring. Choffnes *et al.* [14] studied transgene integration patterns of several hundred plants in subsequent generations. It was demonstrated that the original transgene integration patterns of regenerated primary transgenic plants (T_0_) were not stable when passed to progenies, possibly due to a high level of homologous recombination. Another study performed cytogenetic analyses of GM maize and revealed a fragile phenotype of 45S rDNA as a consequence of genetic modification [15]. McCabe *et al.* [11] detected differences in transgene expression between T_0_ plants and plants grown from selfed T_0_ seeds (T_1_ generation) of transgenic lettuce. And it was suggested that these differences were due to inactivation or suppression of transgene expression. Ulian *et al.* [16] reported instabilities of transfer DNA (T-DNA) insertions in T_0_ plant genomes of petunia. Differences in plant protein expression that originated from different locations were also discovered [17,18].

In recent years, new methods have been adapted for profiling the epigenome, proteome, and transcriptome of GMOs [19]. La Paz, *et al.* [20] analyzed cytosine methylation of the MON810 transgene in different MON810 varieties. The methylation level of the transgene was very low and a comparison between the different varieties revealed no significant differences in symmetric DNA methylation. In contrast, significant differences were observed in the asymmetric sites that play a minor role in epigenetics. Most of the reported comparative transcriptome analyses of MON810 maize were performed using microarrays [21,22,23]. La Paz *et al.* [24] analyzed MON810 and isogenic maize varieties using high-throughput RNA sequencing and found 140 differentially expressed genes. The authors suggested that the differences were due to a slightly delayed maturation process of MON810 compared to the conventional varieties. A proteomic approach was performed by Agapito-Tenfen *et al.* [25]. Using two-dimensional gel electrophoresis combined with mass spectrometry, 32 differentially expressed proteins were identified in Brazilian MON810 maize when compared to isogenic control maize. Rang *et al.* [26] detected different RNA variants transcribed from the transgene in Roundup Ready soybean which may code for 5-Enol-pyruvylshikimate-3-phosphate synthase (EPSPS) fusion proteins.

According to the European Food Safety Authority (EFSA) guidance document and the EU directive 2001/18/EC, the introduced DNA construct must be stable and no changes in the DNA construct should occur during the cultivation and propagation of the plants [27,28]. Even minor changes in the construct are critical because they may lead to unintended changes in the plant properties, content, and/or morphology [29]. Additionally, genetic heterogeneity in the samples may render the analysis for GMO detection unreliable or equivocal [30]. The EFSA guidance document details the requirements for the authorization of GMOs. Accordingly, the GMO events must be analyzed for DNA rearrangements and instabilities of the insert [27,28]. Genetic stability is commonly verified by Southern Blot analysis, however, Southern Blots have drawbacks, including low sensitivity (e.g., only major DNA changes can be detected). In many cases minor changes may occur, which can have an impact on the plant. A variety of methods have been used for analyzing minor nucleotide changes. Genomic samples can be sequenced directly or GMOs can be analyzed by methods such as Sensitive Capillary Electrophoresis (CSCE) or long-range polymerase chain reaction (PCR). Further, real-time PCR employing Scorpion primers for amplification or high resolution melting (HRM) analysis can be used to distinguish between different alleles or to filter out mutated samples [31,32].

According to EFSA, applicants must demonstrate the genetic stability of the transgenic locus over five generations or vegetative cycles before a product can be authorized. For stacked events, the applicant must establish the integrity of the inserts [33]. Further, EFSA determined that the risk assessment of stacked events consisting of events approved by the EU should focus on the genetic stability of such plants, the expression level of the transgenes, and unintended interactions between the stacked events [34]. The genetic stability of the single insert is verified by Southern Blot and segregation analyses before authorization [4,35,36]. The EU directive 2001/18/EC requires that the GMOs be inspected every 10 years [28]. Aguilera *et al.* recommended the validation of the genetic stability of inserts for the whole lifespan of a product [37].

In this study, the genetic stability of a stacked maize (MON88017 × MON810) and that of a single oilseed rape event (GT73) were investigated. MON88017 × MON810 is a maize variety produced by conventional crossing of a double-stacked maize (MON88017) that expresses a *CP4 EPSPS* gene from Agrobacterium sp. CP4, conferring resistance to glyphosate, and the coleopteran-active delta-endotoxin Cry3Bb1 gene from Bacillus thuringiensis (Bt) (*cry3Bb1*) to provide protection against coleopteran insects, particularly the maize rootworm, and the lepidopteran-active delta-endotoxin Cry1Ab (*cry1Ab*)-containing MON810 for protection against lepidopterans such as *Ostrinia nubilalis* and *Sesamia* spp. GT73 is a glyphosate-tolerant oilseed rape [38]. We used real-time PCR with Scorpion primers for amplification (Scorpion PCR) and HRM analysis to examine whether DNA alterations occurred amongst individual seeds (of MON88017 × MON810 maize) or plants (of GT73 oilseed rape). Earlier studies have adapted these methods to analyze the genetic stability and organization of transgenic insertion events. In Neumann *et al.* [32] it could be shown that single nucleotide polymorphism (SNPs) in plasmids can be demonstrated by Scorpion analysis. As a further work for optimizing the method, different alleles of the alcohol dehydrogenase 1 (*ADH1*) gene in maize were investigated by Madi *et al.* [31]. In single seeds, the homozygous as well as heterozygous state of the *ADH1* gene was found. Alleles can be discriminated using Scorpion PCR as well as by HRM analysis. They can be separated by cloning the PCR-amplified DNA. Testing of these clones allows a better differentiation between the alleles compared to testing only the genomic DNA.

In the present work, real-time PCR in combination with subsequent HRM analysis is used as a highly sensitive mutation scanning of selected DNA regions as a standardized means for determining the level of the genetic stability. Samples that potentially carried mutations were cloned into plasmids and sequenced by the Sanger method to verify the DNA alterations.

## 2. Results and Discussion

### 2.1. Basics of Screening Genetically Modified Organisms (GMOs) for Genetic Stability Using Scorpion Polymerase Chain Reaction (PCR) and High Resolution Melting (HRM) Analysis

We analyzed border regions between the endogenous plant genome and the exogenous transgene, known as “event-specific” regions. These regions are being used for the official GMO control. Unidentified mutations may render the test results unreliable. Therefore, we examined the border regions for establishing the genetic stability of GT73 in oilseed rape and MON810 in MON88017 × MON810 maize.

In contrast to the earlier investigations on MON810 [20,32], our study examined MON810 in a stacked event. Combinations of transgenic traits increase the complexity of risk assessment because more undesired scenarios may occur. At first the independent single events present in the stacked event should be safe and stable. In addition, interactions between the events as well as synergistic effects need to be analyzed [33]. Recent studies have demonstrated the suitability of real-time PCR with Scorpion primers and HRM analysis for investigating the nucleotide polymorphisms in genetically modified plants. These tests can be performed readily and are cost effective. The characterization of the Scorpion primers used for the analysis of GMOs has been described by Neumann *et al.* [32] and Thelwell *et al.* [39]. Madi *et al.* [31] have shown that Scorpion PCR combined with HRM analysis is a highly sensitive method for the detection of DNA alterations, including SNPs. Several studies have demonstrated the functionality of HRM analysis [40,41].

The potential genomic alterations were analyzed via a two- to three-step screening procedure, followed by sequencing. In the first step, a large number of individual seeds (MON88017 × MON810 maize samples) or leaves (GT73 oilseed rape samples) were tested using either real-time PCR with Scorpion primers or HRM analysis. The results of this step helped identify the samples that might harbor a genetic divergence. High *C*_t_ values obtained from Scorpion PCR analysis and low confidence values obtained from HRM analysis suggest a modification (SNP or other small sequence alterations) in the target region of the sample (see supplementary Figures S1–S4). Values from the first screening tests were used to select samples for a second round of screening with a lower number of genomic samples. Madi *et al.* have shown that PCR-cloning can be used to separate alleles from genomic samples containing heterozygous mutations [31]. Therefore, in the subsequent screening step, the same primers were used to clone the PCR fragments of the selected samples into plasmids. These plasmids were subjected to HRM analysis alone or HRM analysis and Scorpion PCR. The results from the last screening were used to filter out plasmids that harbor mutations. However, the HRM or Scorpion analysis of genomic samples or plasmids could yield false positive results due to variance among samples or because of variations in DNA quality. With the screening procedure alone, the number of true positive samples could not be determined. To avoid such artifacts and accurately identify samples that harbor alterations, the selected samples from the screening were subjected to sequencing as a secondary validation method.

### 2.2. Screening of 5' Flanking Region of the GT73 (5'-GT73)

**Figure 1:**
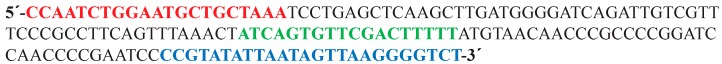
Illustration of the target sequence of the Scorpion primers and the High Resolution Melting (HRM) analysis employed for examining the 5' flanking region of the GT73 (5'-GT73) transgene. The forward primer is shown in red, and the blue letters indicate the reverse primer. The probe sequence (green) lies exactly along the transition from the transgene to the oilseed rape genome. The analyzed sequence contained 156 nucleotides.

The screening of the 5' junction of the oilseed rape variety was performed using a three-step procedure employing real-time PCR with Scorpion primers and HRM analysis. The analyzed target sequence of the 5'-GT73 region is shown in Figure 1. The primer sequences are marked in color and are additionally described in Table 1.

**Figure 2:**
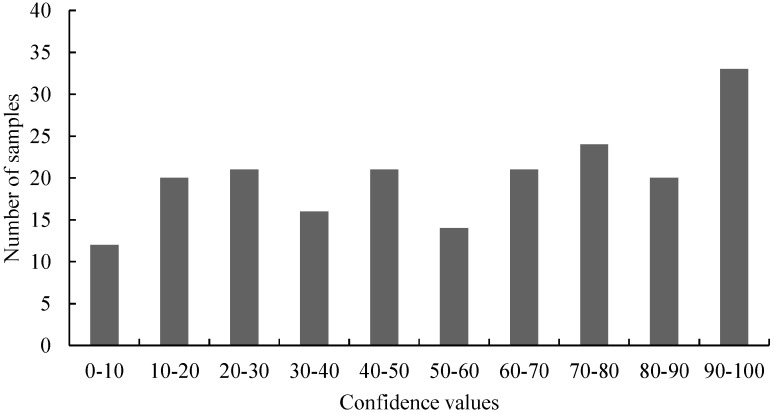
The distribution of the HRM measurements of 5'-GT73 oilseed rape tested by HRM analyses. For the HRM analysis, the confidence values (range: 0.67–99.67) were divided into 10 classes. The values of these classes are on the *x*-axis and the number of samples for the classes on the *y*-axis.

In total, 202 individual GT73 oilseed rape samples were tested by real-time PCR employing Scorpion primers and by HRM analysis on three different days. The *C*_t_ values ranged from 38.01 to 51.84. The average *C*_t_ value and the standard deviation were 42.78 ± 3.80. A pool of 20 genomic GT73 oilseed rape samples (5'-GT73 pool) served as reference to calculate the confidence values. Further, the confidence values ranged from 0.67 to 99.67, with an average value and a standard deviation of 55.44 ± 29.44. The distribution of the confidence values of the first screening was calculated according to Madi *et al.* [31]. Figure 2 shows the graphical distribution of the HRM measurements.

**Table 1:** Sequences of primers used for real-time PCR screening and for PCR cloning.

Primer	Sequence
3m810fw	5'-CCAAGCACGAGACCGTCAA-3'
3m810rev	5'-GCTCGCAAGCAAATTCGGAA-3'
VW01 [ 41]	5'-TCGAAGGACGAAGGACTCTAACG-3'
VW03 [ 41]	5'-TCCATCTTTGGGACCACTGTCG-3'
5GT73fw	5'-CCAATCTGGAATGCTGCTAAA-3'
5GT73rev	5'-AGACCCCTTAACTATTAATATACGG-3'
5GT73Scorprev	5'-[F]AAAAAGTCGAACACTGAT[Q][HEG]AGACCCCTTAACTATTAATATACGG-3'
3GT73fw	5'-CCATATTGACCATCATACTCATTGCT-3'
3GT73rev	5'-GCTTATACGAAGGCAAGAAAAGGA-3'
M13fw lo2	5'-AACGACGGCCAGTGAATTGTAATACG-3'
M13rev lo2	5'-CAGGAAACAGCTATGACCATGATTACG-3'

[F] Fluorophore, [Q] quencher, [HEG] stopper, the F/Q of 5GT73Scorprev are Cy5/BHQ1.

A total of 74 samples from the first round of screening showing high Scorpion *C*_t_ values or low confidence values were selected for further screening. In this screening step, confidence values of the HRM measurements and the *C*_t_ values of the Scorpion analysis were calculated. The *C*_t_ values varied between 37.69 and 46.44, with an average value and a standard deviation of 42.00 ± 1.97. For calculation of the confidence values the 5'-GT73 pool was used again. The confidence values ranged from 2.86 to 99.67. The average confidence value and the standard deviation were 62.29 ± 30.12. Based on the results of the first screening, eight samples with high *C*_t_ values and five samples with low confidence values as observed in the HRM analysis were selected for the second screening. Additionally, two control samples with average *C*_t_ and confidence values were determined. The data of the selected samples are presented in Table 2 (additional information of Table 2 is provided as supplementary file (Table S1). In the supplementary table, all *C*_t_ and confidence values of selected samples are presented).

**Table 2:** (**a**) After the first screening, eight samples (13, 14, 18, 20, 30, 40, 152 and 170) were chosen for further analysis based on the *C*_t_ values. Sample 13 showed the highest *C*_t_ value; (**b**) Six samples (137, 152, 153, 156, 157 and 333) were selected because of low confidence values. Sample 152 was chosen because of high *C*_t_ and very low confidence values; (**c**) The two control samples, 118 and 175, with average *C*_t_ and confidence values are also shown.

(a)	Sample	*C*_t_ Value	(b)	Sample	Confidence Value	(c)	Sample	*C*_t_ Value	Confidence Value
	13	51.84		137	11.80		118	40.54	35.13
	14	47.64		152	2.86		175	41.40	37.49
	18	48.30		153	9.17				
	20	45.77		156	9.11				
	30	45.93		157	17.55				
	40	46.67		333	10.15				
	152	46.57							
	170	45.11							

Based on the results of Madi *et al.*, we assumed that nucleotide changes could also occur as heterozygous alleles [13]. Therefore, prior to the second screening, the selected samples were cloned into plasmids using PCR-cloning (see Experimental Section) to characterize the possible heterozygous alleles. From each selected sample (Table 2), two to three plasmids were generated and the DNA preparations were diluted to a concentration of 10^−^^4^ ng/µL. These were tested in the second round of screening by real-time PCR employing Scorpion primers and were subsequently subjected to HRM analysis. A plasmid pool consisting of 20 plasmids served as reference. The results of the second screening are shown in Table 3.

**Table 3:** Illustration of the Scorpion *C*_t_ and HRM confidence values obtained from the second round of screening using plasmids.

Sample	*C*_t_ Value	Confidence Value	Sample	*C*_t_ Value	Confidence Value
13-2	27.40	99.51	137-2	29.12	94.98
13-3	29.33	98.45	137-3	30.67	99.02
14-1	28.50	98.49	152-1	38.42	90.38
14-3	28.26	98.11	152-2	29.33	99.04
18-1	28.02	94.65	152-3	28.36	86.10
18-2	28.64	92.18	153-1	29.30	99.18
20-1	32.16	99.51	153-2	28.07	93.83
20-2	34.58	95.70	153-3	29.19	96.05
20-4	32.45	10.07	156-2	29.92	88.81
30-2	31.12	70.30	156-3	27.35	97.89
30-3	29.89	94.64	157-2	33.44	81.05
30-5	29.58	97.83	157-3	27.74	98.92
40-1	32.92	4.60	170-2	31.27	95.93
40-2	32.41	99.42	170-3	31.36	25.01
40-3	32.42	93.27	175-2	31.44	16.65
118-1	29.21	98.37	175-3	28.48	0.00
118-2	24.60	97.01	333-1	28.52	93.82
118-3	27.89	99.58	333-2	38.83	78.62
137-1	29.09	97.96			

Because plasmids with low confidence values (<50) could possibly contain SNPs, samples 20-4, 40-1, 170-3, 175-2, and 175-3 were selected for sequencing. Sequencing results showed no genetic instabilities. To exclude the possibility that the low number of plasmids generated per genomic sample prevented the identification of heterozygous DNA samples, we generated 30 more plasmids from the genomic samples 40, 175 and 333 (10 plasmids per sample). These plasmids were tested by Scorpion PCR and HRM analyses. The above-mentioned plasmid pool served as reference again. Results of this screening are depicted in Table 4.

As shown in Table 4, the *C*_t_ values ranged from 20.69 to 23.44 and the confidence values ranged from 69.21 to 99.79. With both, *C*_t_ values and confidence values, the differences between samples were very low. Nevertheless, a minimum of one plasmid per group was sequenced. However, differences in the nucleotide sequence were not detected. Thus, real-time PCR and HRM analyses revealed no genetic instabilities in the analyzed 5' flanking region of the GT73 oilseed rape.

**Table 4:** Scorpion *C*_t_ and HRM confidence values obtained from the screening of additional plasmids (containing 5'-GT73 region). For each genomic sample, 10 cloned fragments were obtained (differentiated by letters a to k).

Sample	*C*_t_ Value	Confidence Value	Sample	*C*_t_ Value	Confidence Value
40-a	23.44	99.64	175-f	21.25	97.37
40-b	22.08	97.46	175-g	22.31	84.30
40-c	22.25	95.40	175-h	21.16	90.70
40-d	22.78	98.56	175-i	21.08	98.68
40-e	22.32	96.64	175-k	23.05	98.79
40-f	21.99	97.56	333-a	22.25	99.79
40-g	22.40	99.00	333-b	22.15	97.95
40-h	22.10	98.95	333-c	22.30	96.00
40-i	22.14	95.38	333-d	21.77	91.31
40-k	22.19	97.35	333-e	21.72	71.89
175-a	21.09	99.26	333-f	22.27	85.75
175-b	21.02	96.66	333-g	21.88	85.34
175-c	23.38	98.21	333-h	21.60	85.15
175-d	22.05	93.81	333-i	21.70	99.57
175-e	21.30	97.86	333-k	20.69	69.21

### 2.3. Screening of 3' Flanking Region of the GT73 (3'-GT73)

**Figure 3:**
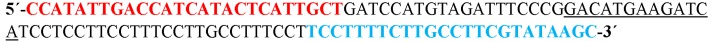
Illustration of the target sequence used in the HRM analysis of the 3' junction of GT73 transgene. The transgene sequence is at the 3' region, and the transition to the genome lies in the underlined region. The forward primer is shown in red and the blue letters indicate the reverse primer. The sequence contains 108 nucleotides.

Although we attempted to use Scorpion PCR followed by HRM analysis to screen the 3' flanking region of the GT73 transgene for mutations, we were unable to develop an adequate Scorpion primer with the desired elements. Specifically, we were unable to distinguish a wild-type plasmid from a plasmid with a 2-bp deletion. Therefore, the first screening was performed using HRM analysis alone. A 108-bp nucleotide sequence at the 3'-GT73 flanking region was analyzed using real-time PCR and HRM analyses. Figure 3 shows the 3'-GT73 target sequence. The primer sequences are presented in Table 1.

All in all, 510 individual samples were screened by HRM analysis. The average confidence value and the standard deviation were 55.16 ± 33.60. A pool of 20 genomic GT73 oilseed rape samples (3'-GT73 pool) served as reference to calculate the confidence values. The distribution of the confidence values was also calculated. The confidence values of the screening were divided into 10 groups and the cluster frequencies were determined.

**Figure 4:**
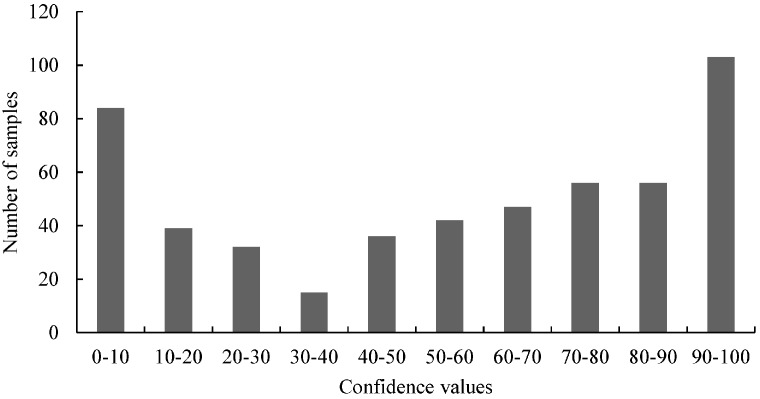
The distribution of the HRM measurements of 3'-GT73 oilseed rape. For the HRM analysis, the confidence values (range: 0.00–99.97) were divided into 10 classes. The values of these classes were drawn on the *x*-axis and the number of samples for the classes on the *y*-axis.

As illustrated in Figure 4, the clusters with the lowest and the highest confidence values had the highest frequencies. The distribution of the 3'-GT73 confidence values was congruent to a bimodal sample pool containing wild-type (confidence values > 90) and mutant (confidence values < 10) samples. For further investigations, 47 samples with low confidence values were selected and screened two times using HRM analysis. The genomic 3'-GT73 pool served as reference. Based on the average values of this screening, 10 samples with the lowest confidence values were selected (Table 5).

**Table 5:** The samples 50, 51, 63, 70, 72, 99, 145, 379, 388 and 494 were chosen based on the confidence values of the second screening. Sample 63 had the lowest confidence value.

Sample	Confidence Value
50	1.31
51	1.69
63	0.94
70	4.14
72	1.00
99	3.16
145	10.44
379	10.05
388	25.31
494	55.22

These genomic samples were cloned into plasmids by PCR-cloning (Zero Blunt TOPO PCR Cloning kit). For the PCR, a high-fidelity proofreading enzyme (Phusion Hot Start II DNA Polymerase) was used and the same primer pair employed for the first 3'-GT73 screening was utilized. One or two plasmids were generated per selected genomic sample. Plasmid preparations were diluted to a concentration of 10^−4^ ng/µL and analyzed in a second screening step using the 3'-GT73 primer pair. A pool of 10 plasmids served as reference. The results of the second screening are shown in Table 6.

**Table 6:** In the second screening, 17 plasmids were analyzed. The confidence values ranged from 86.96 to 99.92.

Sample	Confidence Value
50-3	92.04
50-4	99.92
51-1	95.27
51-2	97.84
63-1	86.96
70-1	96.85
72-1	89.00
72-2	99.54
99-2	98.34
99-3	98.83
145-1	94.89
145-2	98.18
379-3	96.40
379-4	99.78
388-1	99.67
494-1	99.81
494-2	99.78

The confidence values obtained for the plasmids in the second screening are different from those of the first screening with genomic samples. Whereas the confidence values of the selected samples in the first HRM screening were low (eight samples below 11 and two samples below 60; Table 5), the HRM analysis of the second screening yielded very high confidence values (>85). Based on these results, we concluded that none of the samples had a mutation in the targeted region. To verify this, two plasmids were sequenced. Sequencing confirmed that mutations were absent. Thus, the analysis of the 3' flanking region of the GT73 transgene in oilseed rape leaves revealed no genetic instabilities.

### 2.4. Screening of 5' Junction of MON810 (5'-MON810)

**Figure 5:**
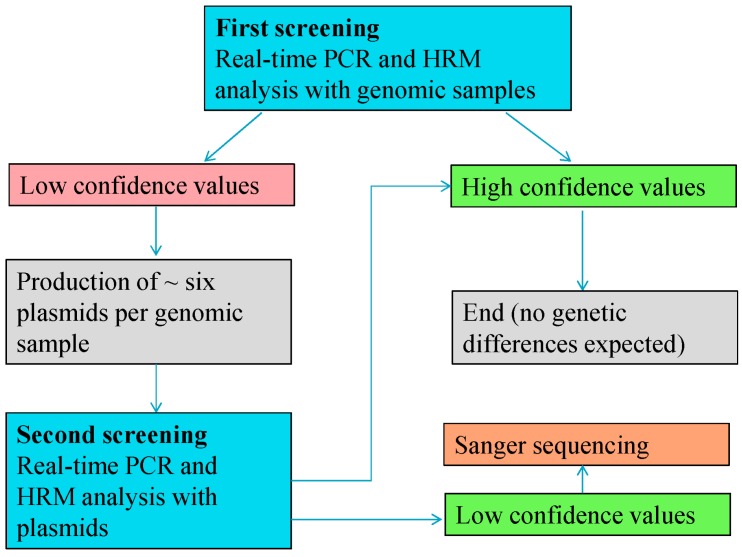
The two-step screening schema for the analysis of MON810. Only samples with low confidence values in the first screening were selected for the second screening. Samples with high confidence values were associated with wild-type and were not analyzed. Approximately six or more plasmids were generated per selected genomic sample. After the final screening step, selected plasmids were sequenced to identify DNA alterations, and the type and the exact location of the mutations were determined.

For the investigation of 5' junction of MON810 (5'-MON810), a stacked GM maize variety (MON88017 × MON810) containing three genetic elements was used. The analyzed target region has been described by Neumann *et al.* [32] and the primers are shown in Table 1. It was possible to test this region using Scorpion PCR [32] and HRM analysis. However, because Scorpion analysis only reveals DNA alterations in the probe and primer regions and HRM analysis identifies changes in the whole target sequence, only HRM analysis was performed. For the screening of the 5'-MON810 region, a two-step procedure was employed. In the first step, individual genomic samples of seeds were tested using real-time PCR and HRM analyses. In the second step, the genomic samples that showed the possible presence of mutations were cloned into plasmids and examined by HRM analysis. Finally, these plasmids were sequenced to identify possible mutations. Figure 5 gives an overview of the screening schema.

**Figure 6:**
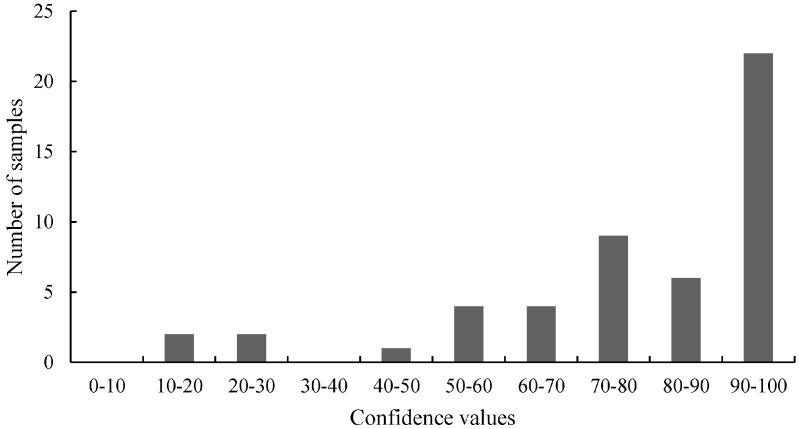
The distribution of the HRM measurements of the 5' junction of MON810 (5'-MON810) region tested with real-time PCR. For the HRM analysis the confidence values were divided into 10 classes. The values of these classes were plotted on the *x*-axis and the numbers of the samples for the classes on the *y*-axis.

In the first step, 50 maize samples (MON88017 × MON810) were tested twice (separately on different days). A pool of 20 genomic maize samples (MON88017 × MON810) served as reference to calculate the confidence values. Figure 6 shows the graphical distribution of the HRM measurements. If the samples harbored mutations, a distribution similar to that in Figure 4 would be expected and very high (wild-type) and very low (mutants) confidence values would be very frequent. However, this was not observed. Cluster 10 showed the highest frequency, whereas cluster one had a very low frequency. The confidence values ranged from 13.16 to 99.83. The average confidence value and the standard deviation were 77.88 ± 23.36.

Samples with confidence values below 10 were not present. Four samples, which had confidence values below 25, were chosen for the second screening. Additionally, sample 383 was selected because a pre-screening revealed that this sample contained an SNP in the coding region of the MON810 transgene (see Section 2.5.). The selected samples and the confidence values of the first 5'-MON810 screening are shown in Table 7.

**Table 7:** Four samples, 272, 275, 322 and 354 were chosen based on the confidence values of the first screening. Sample 383 was selected because it showed a DNA alteration at the 3'-MON810 junction.

Sample	Confidence Value
272	22.41
275	23.18
322	15.95
354	13.16
383	75.80

The 5'-MON810 target region of the chosen maize samples was cloned into plasmids by PCR-cloning (see Experimental Section). Results from our analysis of GT73 suggested that a high number of clones are necessary to distinguish the authentic mutations from artifacts. Therefore, six plasmids with cloned PCR fragments were obtained for each sample. Thus, a total 30 plasmids were obtained and tested in the second 5'-MON810 screening round using HRM analysis employing the same primer pair as that used for the first screening. The confidence values ranged from 17.88 to 99.94. The average value and the standard deviation were 95.02 ± 15.15. Table 8 shows the confidence values of the second screening.

**Table 8:** Plasmid 272-5 served as reference (confidence value of 100). Plasmids 275-8 and 272-4 showed the lowest confidence values.

Plasmid	Confidence Value	Plasmid	Confidence Value
272-2	99.66	322-4	90.11
272-3	99.94	322-5	96.53
272-4	79.48	322-6	99.14
272-5	100.00	354-1	99.75
272-6	99.62	354-2	99.17
272-8	99.23	354-4	91.91
275-1	96.43	354-5	99.56
275-2	95.71	354-7	99.55
275-3	98.19	354-8	99.62
275-5	98.91	383-2	97.87
275-6	99.68	383-3	96.63
275-8	17.88	383-4	98.40
322-1	99.88	383-5	99.63
322-2	98.81	383-6	99.67
322-3	99.71	383-7	99.92

As shown in Table 8, only plasmid 275-8 had a low confidence value (17.88). Plasmid 272-4 showed the second lowest value (79.48). All other plasmids showed a confidence value of >90 and therefore were associated with the wild-type. To verify if the plasmids 275-8 and 272-4 harbored mutations, these samples were subjected to Sanger sequencing. The sequencing revealed an SNP, a C→T substitution, at position 147 in plasmid 275-8, but not in 272-4. Because no other plasmid generated from the sample 275 showed this DNA alteration, this SNP was assessed as due to polymerase error. Polymerase errors can occur even when high-fidelity enzymes are used. The theoretical probability of having a polymerase error in the present cloning sequence was 0.003 (calculated according to Foissac) [42]. Thus, three out of 1000 fragments may contain an error. The calculated error frequency is markedly lower than that expected based on the results of our experiments. A somatic mutation can also be the reason for the altered DNA sequence of sample 275-8. However, false positive results demonstrate the importance of the cloning step and the subsequent detailed characterization of these plasmids after the first screening.

### 2.5. Screening of 3' Junction of MON810 (3'-MON810)

**Figure 7:**
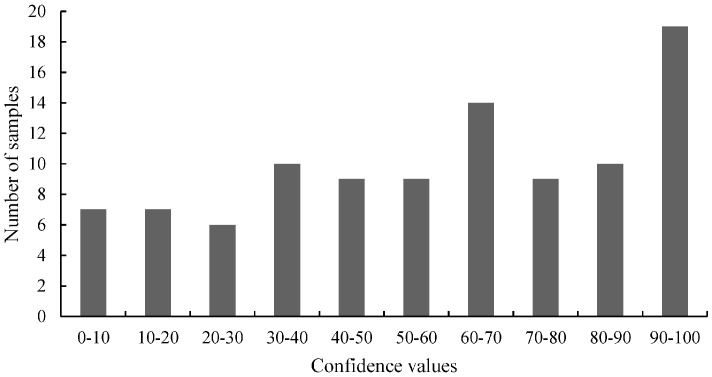
The distribution of the HRM measurements of the 3' junction of MON810 (3'-MON810) region tested with real-time PCR. For the HRM analysis the confidence values were divided into 10 classes. The values of these classes were plotted against the number of samples.

We analyzed the 3' junction of MON810 (3'-MON810) region of the GMO variety MON88017 × MON810. The 3'-MON810 target sequence has been reported previously by Neumann *et al.* [32]. The primers are shown in Table 1. The two-step procedure shown in Figure 5 was used for screening. The distribution of the HRM measurements is shown in Figure 7. Based on this distribution, no or very few mutations would be expected. This is because a bimodal distribution was not evident. A pool of 20 genomic maize samples (MON88017 × MON810) served as reference to calculate the confidence values. The confidence values ranged from 0.54 to 99.03. The average confidence value and the standard deviation were 57.91 ± 29.59.

For the second screening, eight samples from clusters one and two were chosen. Table 9 shows the selected samples and the confidence values obtained from the HRM analysis performed for the first screening.

**Table 9:** The eight samples shown were selected for the second screening because they resulted in low confidence values in the first screening.

Sample	Confidence Value
263	10.95
264	1.23
345	0.54
352	7.91
354	8.45
355	3.01
374	10.88
383	4.81

The 3'-MON810 target regions of the chosen maize samples were cloned into plasmids (see Experimental Section). For each sample, six plasmids were obtained and are shown in Table 9. Thus, in the second 3'-MON810 screening, 48 plasmids were subjected to HRM analysis using the same primer pair as that employed for the first screening. The confidence values ranged from 2.94 to 99.14. The average value and the standard deviation were 70.46 ± 33.69. Table 10 shows the confidence values obtained from the second screening.

**Table 10:** A total of 48 plasmids (containing the 3'-MON810 target region) were analyzed in the second screening. The plasmid 383-2 served as reference (confidence value of 100).

Plasmid	Confidence Value	Plasmid	Confidence Value
263-2	67.69	354-1	44.77
263-3	72.02	354-2	15.97
263-4	66.57	354-3	90.48
263-5	62.97	354-4	64.39
263-7	82.78	354-5	62.92
263-8	88.24	354-6	81.43
264-1	88.51	355-1	91.75
264-2	7.99	355-2	90.81
264-3	4.61	355-3	89.48
264-4	4.95	355-4	95.72
264-6	6.01	355-5	94.10
264-7	7.97	355-7	94.76
345-2	64.71	374-2	99.14
345-3	75.71	374-3	99.01
345-4	78.59	374-4	98.39
345-5	86.28	374-6	98.75
345-6	91.02	374-7	98.61
345-7	84.85	374-8	96.73
352-1	81.06	383-1	3.14
352-2	96.06	383-2	100.00
352-3	95.95	383-5	97.56
352-4	97.95	383-7	95.53
352-5	93.88	383-8	6.40
352-6	92.63	383-9	2.9

**Figure 8:**
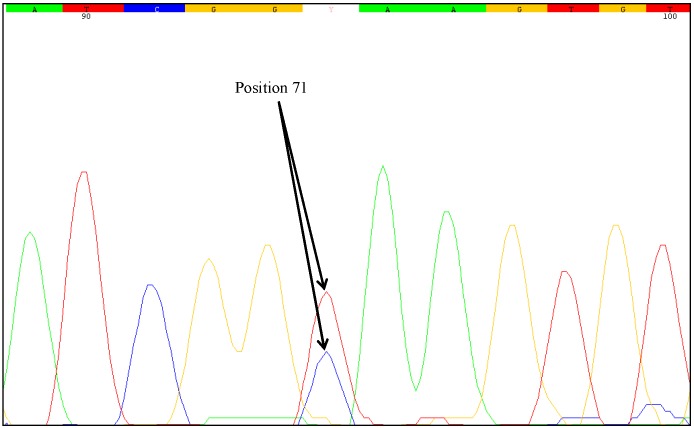
The sequencing chromatogram of the 3'-MON810 target region of sample 264 clearly showed a double peak at position 71. Double peaks occur when different alleles are present.

Several plasmids were chosen for sequencing. These are depicted in Table 11. Altered nucleotide sequences were detected in 14 plasmids. These changes were verified by forward and reverse sequencing. The plasmids with altered nucleotide sequences belonged to three different genomic samples, 264, 354 and 383. In the case of sample 264, six out of seven plasmids showed a C→T substitution at position 71 of the analyzed 3'-MON810 target sequence, whereas four of seven plasmids of sample 383 showed a C→T substitution at position 71. A heterozygous SNP appeared to be present in sample 383. For sample 264 it was not clear whether the SNP was homozygous or heterozygous. Thus, the PCR product of the 3'-MON810 target region in sample 264 was subjected to direct sequencing. As shown in Figure 8, the result indicated that the SNP was heterozygous. In the sequencing chromatogram, an overlay of two peaks was present at position 71. A thymine peak was found to overlay a cytosine peak. Direct sequencing of the 3'-MON810 target region of the sample 383 also revealed an overlay of a cytosine and a thymine peak (data not shown). The results for sample 354 favor the notion that this was a false positive. This is because there were many different types of SNPs. In addition to the wild-type three different substitutions (G→T, C→T und C→A) at three different positions were found in the plasmids produced from the same genomic sample 354. Direct sequencing of a PCR product of the 3'-MON810 region of sample 354 showed wild-type. Although a plant cultivated from sample 354 would have provided useful information, this approach was not feasible because the whole maize kernel was homogenized for DNA extraction.

It is unknown whether the partially triploid state of the grain may have influenced the results. Endosperm, a triploid tissue, accounts for 80%–90% of the maize kernel’s weight. Endosperm contains two maternal and one paternal genome copies. For interpreting the results, it is important to consider the degree of zygosity of the analyzed maize variety. It makes a difference if the seeds are hemi- or homozygous for the transgenic locus and in the case of hemizygosity, it is important to know if the transgene donor is male or female [43]. The degree of zygosity in the analyzed maize variety (MON88017 × MON810) remains unknown.

**Table 11:** Overview of the sequencing results for 3'-MON810 plasmids. Plasmids sequenced because of the screening results are marked with *. All other plasmids were sequenced subsequently because of the prior sequencing results. The confidence values are shown together with sequencing results for a better overview.

Plasmid	Confidence Value	SNP	Position
263-2	67.69	no	
263-3	72.02	no	
263-4	66.57	no	
263-5	62.97	no	
263-7	82.78	no	
263-8	88.24	no	
264-1	88.51	no	
264-2 *	7.99	C→T	71
264-3 *	4.61	C→T	71
264-4 *	4.95	C→T	71
264-6 *	6.01	C→T	71
264-7 *	7.97	C→T	71
264-9	not tested	C→T	71
345-2	64.71	no	
345-3	75.71	no	
345-4	78.59	no	
345-6	91.02	no	
352-1	81.06	no	
352-2	96.06	no	
354-1	44.77	G→T	74
354-2 *	15.97	C→T	86
354-3	90.48	no	
354-4	64.39	C→A	84
354-5	62.92	C→A	84
354-6	81.43	no	
355-3	89.48	no	
355-7	94.76	no	
374-2	99.14	no	
374-8	96.73	no	
383-1 *	3.14	C→T	71
383-2	100.00	no	
383-5	97.56	no	
383-7	95.53	no	
383-8 *	6.40	C→T	71
383-9 *	2.94	C→T	71
383-11	not tested	C→T	71

**Figure 9:**
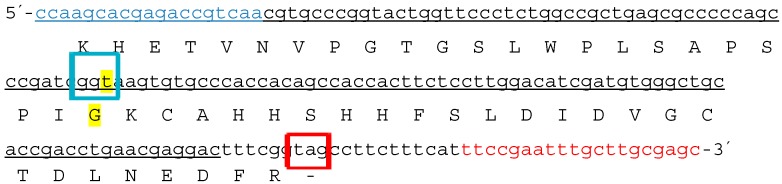
The analyzed 3'-MON810 target region is shown. The underlined DNA sequence belongs to the transgenic construct. The forward primer is shown in blue and the reverse primer in red. The SNP location is highlighted in yellow and the affected codon is framed blue. Cytosine is substituted by a thymine at position 71. The amino acid sequence of the *cry1Ab* gene is shown below the nucleotide sequence. The coded amino acid glycine (marked in yellow) is not affected by the SNP. The stop codon (framed red) lies within the genomic DNA because a truncation event at the 3' end of the *cryIAb* gene led to the loss of the nopaline synthase (NOS) terminator (for more details see [17]).

All SNPs detected in a study by Ogasawara *et al.* were silent mutations [44]. Silent mutations do not result in an altered amino acid sequence. The SNPs at position 71 identified in samples 264 and 383 were also silent mutations (the codons GGC and GGT both code for the amino acid glycine). This is illustrated in Figure 9. The detected SNP lies within the coding region of the *cry1Ab* toxin.

The results showed that a nucleotide change at the 3'-MON810 region was present in two out of 100 analyzed samples. However, the true rate of mutation at the 3' end of the MON810 transgene in MON88017 × MON810 remains unknown. For an estimation of this rate, a higher number of samples need to be analyzed.

## 3. Experimental Section

Unless stated otherwise, the chemicals, PCR reagents, and PCR primers used were of analytical grade and were purchased from Biozym (Hessisch Oldendorf, Germany) (PCR reagents) and Sigma–Aldrich (Vienna, Austria) (PCR primers and chemicals). The Zero Blunt TOPO PCR Cloning kit (Invitrogen, Vienna, Austria), EasyPrep Pro Plasmid Miniprep kit (Biozym), JM109 Competent Cells (>107 cfu/μg, Promega, Mannheim, Germany), Wizard DNA Clean-up System (Promega), Phusion Hot Start II High-Fidelity DNA Polymerase kit (Biozym), and Type-it HRM PCR Kit (Qiagen, Hilden, Germany) were used according to the respective manufacturer’s recommendations. The stacked event MON88017-3 × MON-00810-6 (hybrid 4421VT3) maize was obtained from Croplan Genetics^®^ (Williston, ND, USA). And the GT73 (MON-00073-7) oilseed rape seeds were obtained by taking plants randomly from a field in Saskatchewan, Canada (season 2011). GT73 oilseed rape plants were cultivated in a closed system (in a separate room under controlled conditions: day/night 20 ± 2 °C, 12/12 h light/dark, r. h. 60% ± 5%). Leaves were harvested after four weeks and immediately frozen in liquid nitrogen.

### 3.1. Extraction of Genomic DNA

Maize seeds were homogenized with a household garlic crusher. Plant leaves of GT73 were crushed using a mortar and a pestle after cooling with liquid nitrogen. A total 100 mg of the homogenized leaves (GT73) or 150 mg of the homogenized seeds (MON88017 × MON810) were incubated with a mixture of 820 µL of TNE buffer (10 mM Tris (pH 8), 150 mM NaCl, 2 mM EDTA (pH 8)), 1% SDS, 150 µL of 5 M Guanidine-HCl, and 30 µL of proteinase K (600 µg/mL) overnight at 60 °C under constant shaking. Following this, the mixture was centrifuged for 5 min at 16,100× *g*. To 600 µL of the supernatant, 300 µL of chloroform (99%) was added and the mixture was vortexed for 20 s. The mixture was then centrifuged at 16,100× *g* for 8 min to separate the phases and 500 µL of the aqueous phase was transferred into a new tube. To this, 4 µL of RNAse (32 µg/mL) was added, and the mixture was incubated at 60 °C for 30 min under constant shaking. The extracted DNA was purified using the Wizard DNA Clean-up System (Promega). After a pre-elution with 20 µL of ddH_2_O, the DNA was eluted with 10 mM Tris buffer (pH 7.4, 70 °C) for 10 min. The eluted DNA was immediately stored at −20 °C.

### 3.2. Instruments Used for HRM and Scorpion Analysis

Scorpion PCR experiments can be performed on any standard real-time PCR instrument. For HRM analysis, it is necessary to use a specific cycler optimized for precise melting analysis that uses dedicated heating algorithms and specific software [45,46]. For the Scorpion PCR as well as for the HRM experiments a Rotor-Gene Q (Qiagen) instrument was used. For the calculation of the scores (confidence values) of the HRM analysis, a specific software package (Rotor-Gene 2.0.2.4, Qiagen) of the Rotor-Gene Q cycler was used.

### 3.3. Screening by Real-Time PCR Using Scorpion Primers and HRM Analysis

A total 510 individual oilseed rape samples (GT73) and 100 individual maize samples (MON88017 × MON810) were analyzed on different days by three different operators. The mean of the values was calculated and was used for further investigation. Pools of genomic samples (for primary screenings) or plasmids (secondary screenings) served as reference to calculate the confidence values. The reference served as a control for the performance of all samples (including a positive (standard) control as well as a non-template control), allowed for the verification of the validity of all parameters of the run, and provided a reference value that enabled a comparison of different runs.

Unimolecular, singular Scorpion primers consisting of forward/reverse PCR-primer sequences as well as additional elements were used for this work. The additional elements included a PCR stopper to prevent the PCR read-through of the probe element, and a specific probe sequence with a fluorescence-based detection module consisting of a fluorophore and a quencher. The PCR yields an amplicon containing a sequence that is complementary to the Scorpion probe. The specific probe sequence then binds to its complementary sequence within the amplicon, the hairpin loop opens up, and a signal is produced [32,39].

With HRM analysis, the melting point (*T*m) of the amplicon produced is determined after the PCR reaction. This is achieved by gradually increasing the temperature from the annealing temperature up to 95 °C. Simultaneously, the fluorescence of a DNA intercalating dye is measured. Because high-precision temperature control and fluorescence measurement are required for successfully implementing this method, such measurements can only be made using an HRM-certified equipment [40].

### 3.4. Qualitative PCR for Cloning

The PCR was performed with genomic DNA as template. A total 20 µL of the reaction mixture contained 4 µL of 5× Phusion HF-buffer (proprietary information, 1.5 mM MgCl_2_, error rate was 4.4 × 10^−7^), 500 nM of each primer, 250 µM of each deoxynucleoside triphosphate (dNTP), 0.4 U Phusion Hot Start II DNA Polymerase, and 50 ng of DNA. The temperature profile is shown in Table 12.

**Table 12:** The PCR temperature profile of the two-step protocol used for analysis. Touchdown PCR was used to eliminate non-specific amplification. Therefore, the annealing temperature started at 66 °C and was lowered by 0.5 °C per cycle for the first 18 cycles. The last 22 cycles had an annealing temperature of 60 °C.

Cycle Step	Temp.	Time	Number of Cycles
Initial denaturation	98 °C	2 min	
Denaturation	98 °C	10 s	18
Annealing	66–57.5 °C Touchdown (−0.5 °C each cycle)	30 s	
Extension	72 °C	30 s	
Denaturation	98 °C	10 s	22
Annealing	60 °C	30 s	
Extension	72 °C	30 s	
Final extension	72 °C	7 min	

A 1 µL aliquot of the PCR product was ligated and cloned into pCR-Blunt II-TOPO using the Zero Blunt TOPO PCR Cloning kit (Invitrogen) and transformed into JM109 Competent Cells. Colonies were grown in 5 mL of LB-medium and the recombinant DNA was isolated with the help of EasyPrep Pro Plasmid Miniprep kit (Biozym). Positive colonies were analyzed for the correct length of inserts using agarose gel electrophoresis on 2.5% gels. The validity of the insert was then verified by sequencing using M13 primers.

### 3.5. Real-Time PCR and HRM Conditions

Real-time PCR was performed on a Rotor-Gene Q (Qiagen) instrument. The total volume of the reaction mixture was 16 µL. The Type-it HRM PCR Kit (Qiagen), 700 nM primers (Scorpion primer 125 nM), and 1.6 µL of undiluted DNA (70–100 ng/µL) template were used for each reaction. The thermal cycling profile used is shown in Table 13.

**Table 13:** The thermal cycling profile of the real-time PCR (prior to HRM analysis) experiment.

Cycle Step	Temperature	Time	Number of Cycles
Initial Denaturation	95 °C	5 min	
Denaturation	95 °C	10 s	55
Annealing	55 °C	30 s	
Extension	72 °C	20 s	

The experiments were performed on different days and the mean of the values was calculated and was used for further analysis. HRM was performed with the temperature increasing at a rate of 0.2 °C per 4 s. The initial and final temperatures were 70 and 95 °C, respectively. The melting curves and the temperature-shifted curves were normalized to enable the comparison of samples. Modified curves and HRM scores were obtained using the Rotor-Gene Q series software (version 2.0.2.4, Qiagen). The normalized and temperature-shifted melting curves corresponded to the final curve after normalization. When an amplicon harbored a sequence variation, the normalized and temperature-shifted melting curves had a different shape from those of the wild-type amplicons (see Supplementary Figures S3 and S4).

### 3.6. Sequencing

All sequence analyses were performed using the Sanger sequencing method. The BigDye Fast cycle sequencing protocol, described by Platt *et al.* [47], was used for this purpose. The M13 sequences necessary for sequencing were present in the plasmids generated, as the cloning vector contained these sequences. To avoid sequencing errors, both forward and reverse sequencing were included in the method. This insured the reliability of the sequencing results.

### 3.7. Primers

Primers used for this work are shown Table 1.

## 4. Conclusions

The objective of the present work was to identify DNA alterations that may cause genetic instability in GMOs, particularly in stacked events. The number of mutated samples we found in our study was two (out of 100 stacked maize samples tested). A much higher number of samples was analyzed (*n* = 567 MON810 maize samples and *n* = 1034 RRS 40-3-2 samples of soy) in earlier studies that found no DNA alterations in soybean and maize [31,32]. The SNP frequency we observed in our study markedly exceeds the natural mutation rate. The average single-base substitution rate for maize is estimated to be 5.39 × 10^−8^ per site per year and for genic maize regions, 4.79 × 10^−8^ per site per year [48]. One important point to mention is that the detected SNPs were most likely not identified by low-sensitivity methods such as Southern Blot analysis.

Increased rate of mutation at the border regions may have a negative influence on GMO quantification by real-time PCR. Madi *et al.* [31] concluded that analysis of DNA instabilities in regions commonly used for the quantification of GMOs is particular important. Therefore, in canola and maize, the border regions from the genome into the construct at the 5' and 3' regions of the relevant transgene were tested in our study. Mutations were detected only in the 3'-MON810 region. Therefore, the 5'-MON810 region appears to be more suitable for GMO quantification as no instabilities were identified in this region. Based on the definition that the first base (at the 5' end) of the analyzed 3'-MON810 target region is at position one, we found that both of the mutated samples harbored an SNP at position 71. However, by investigating a higher number of stacked maize samples (MON88017 × MON810), it cannot be excluded that SNPs would be identified at (other) positions which may change the transgenic protein. The inserted 3' flanking region of MON810 is a truncated version of MON810. The 3' end of the *cry1Ab* gene and the nopaline synthase (NOS) terminator were lost during the plant transformation [49]. It is also possible that the occurrence of the SNP detected in this study at the same region was random. One additional aim of this study was to evaluate the impact of the identified SNP on the amino acid sequence. Similar to that reported by Ogasawara *et al.* [44], the identified mutation was silent, meaning the amino acid sequence remained unchanged. Therefore, it is conceivable that the phenotype of the sample was not altered.

One unresolved question is the degree of zygosity of the transgene in the samples examined. Direct sequencing of the PCR products generated from the mutated samples indicated heterozygosity at nucleotide position 71. Two different nucleotides were identified at this position, a cytosine as wild-type and a thymine as mutant-type. The sequencing chromatograms of the PCR products clearly showed an overlay of these bases. However, sequencing errors could lead to similar chromatograms. Some positions may show overlapping nucleotides and it can be difficult to recognize heterozygous mutations just by direct sequencing of the PCR products. Therefore, we used the cloning approach described by Madi *et al.* [31]. Plasmids containing DNA of one allele can be obtained from genomic samples with heterozygous alleles. With clones comprised of one allele, the outcome of sequencing is unambiguous and sequencing error can be ruled out. PCR products of the affected samples were sequenced twice (forward and reverse). The plasmids of the MON88017 × MON810 samples 264 and 383 were also sequenced twice.

In Neumann *et al.* [32] and Madi *et al.* [31] the question was asked if the scores or the distribution of the values of the primary tests, (*i.e.*, the *C*_t_ values of the Scorpion PCR and the confidence values of the HRM analyses) can be used to detect mutations. Our results show that the distribution of the values does not necessarily indicate the presence of mutations (Figure 2, Figure 4, Figure 6 and Figure 7). For samples harboring mutations, two groups can be expected: samples with very high confidence values (wild-type) and those with very low confidence values (mutated samples). However, only Figure 4 (belongs to the distribution of 3'-GT73 samples) showed a distribution consistent with this description and no mutations were identified in these samples. In contrast, the distribution of the values of the 3'-MON810 screening did not show the expected characteristics. However, mutations were found in the 3'-MON810 region.

Based on the mutations identified by this work, we conclude that cloning is required for the unambiguous characterization of SNPs in GMOs. In addition to the two mutations, we also encountered false positive results revealed through subsequent investigation. The different plasmids of the affected sample (354 of MON88017 × MON810 maize) showed inconsistent SNPs (Table 11). Although PCR error is a possible explanation, the precise nature of the factors that contributed to the false positive result is not known. Additionally, plasmid 275-8 of the 5'-MON810 region showed an SNP, which was assessed as due to error in the cloning sequence. A related aspect is the number of plasmids required. Our results show that minimum six to 10 plasmids are necessary for the characterization of heterozygous samples.

Whereas earlier studies investigated single MON810 maize events [20,32], the present work focused on stacked maize event containing MON810. Stacked events having the same promoter are more susceptible to unintended effects of the transgenic event expression [50]. Many stacked events carry transgenes that use the same promoter. The 35S promoter, a viral element, occurs twice in MON88017 × MON810 maize. Kohli *et al.* discussed that the 35S promoter contains a recombination hot spot that has an influence on GMOs [51]. Since stacked events contain multiple viral promoters the susceptibility to instabilities may be increased. Due to an increasing number of stacked events being commercialized, there is a proportionally-relevant need for the analysis of their genetic stability in the context of the specific stacked event in question on a case-by-case basis. Due to the present results one might assume that stacked events tend to be more instable than single events. To confirm this assumption, it would be necessary to demonstrate further instabilities of MON810 in other stacked events. Corresponding experiments are planned. In these, it will be important to analyze whether stacking of the transgenes, and the means by which the stacked varieties were created, may be a contributing factor. It also will be necessary to understand the potential characterization (regulatory) and biosafety issues that may be relevant.

For future studies, it may also be interesting to analyze the zygosity degree of the mutated samples in detail.

## Supplementary Materials

Supplementary materials can be found at http://www.mdpi.com/1422-0067/15/11/19898/s1.
